# VORTEX (Variscite origin recognition technology X-ray based) Data. A European geoarchaeological green phosphate compositional dataset

**DOI:** 10.1016/j.dib.2025.111961

**Published:** 2025-08-06

**Authors:** Daniel Sánchez-Gómez, José Ángel Garrido-Cordero, José María Martínez-Blanes, Rodrigo Villalobos García, Manuel Edo i Benaiges, Ana Catarina Sousa, María Dolores Zambrana Vega, Ferran Borrell, Rosa Barroso Bermejo, Primitiva Bueno Ramírez, Carlos P. Odriozola

**Affiliations:** a UNIARQ, Centro de Arqueologia da Universidade de Lisboa, 1600-214, Lisbon, Portugal; b Dpto. de Prehistoria y Arqueología, Universidad de Sevilla, 41004, Seville, Spain; c Instituto de Ciencia de Materiales de Sevilla, Universidad de Sevilla- Consejo Superior de Investigaciones Científicas, Seville, 41092, Spain; d Cuerpo de Profesores de Enseñanza Secundaria, Gobierno de Cantabria, Spain; e CIPAG (Collectiu per la investigació de la prehistòria i l'arqueologia del Garraf-Ordal),08859, Begues, Spain; f Dpto. de Pintura, Universidad de Sevilla, 41003, Seville, Spain; g CSIC - Institución Mila y Fontanals de Investigación en Humanidades (IMF), 08001, Barcelona, Spain; h Dpto. de Historia y Filosofía Universidad de Alcalá, 28801, Alcalá de Henares, Spain

**Keywords:** Provenance analysis, Machine learning, Random forest, Prehistoric exchange networks, Iberian Peninsula, p-XRF, XRD

## Abstract

This dataset includes the compositional information of *n* = 1778 geoarchaeological samples of green phosphate minerals sourced from three major Iberian deposits with evidence of prehistoric mining: Aliste, Terena, and the Gavà Mines. It has been used to develop a data-driven framework that integrates portable X-ray fluorescence (p-XRF) analysis and machine learning (ML), to determine the provenance of archaeological artifacts based on its elemental composition. In addition, a mineralogical analysis by X-ray diffraction (XRD) of a subset (*N* = 249; n_Aliste = 122, n_Terena = 47, n_Gavà = 80) was conducted to complement the geochemical characterisation of the sample. Data from *n* = 571 variscite beads from 15 Iberian and French archaeological sites, representing a diverse range of chrono-cultural contexts spanning from the Neolithic to the Bronze Age, used to test the model are also presented.

The compositional data was obtained by using a portable XRF analyser and is presented as atomic percentages of the elements between Al and Th. Mineral compositions were determined using a Panalytical X’Pert Pro θ/θ diffractometer. This dataset has high reuse potential for future non-destructive studies of archaeological artefacts and past social network analyses.

Specifications Table

**Table:** 

Subject	Geochemistry, Archaeology.
Specific subject area	Geoarchaeology (Variscite provenance)
Type of data	Table (Raw and Processed data)
Data collection	The chemical composition of the samples was analyzed using an Oxford Instrument XMET-7500 handheld XRF device equipped with a Rhodium (Rh) tube, a silicon drift detector (SDD), and an automatic 5-position filter changer. Quantification was performed using the SOILS-LE program, which employs the fundamental parameter (FP) method. This method is particularly effective for analyzing a large number of elements, making it well-suited for this study. The mean analysis time per sample was 60 s and chemical elements from Mg to Th were measured and quantified. A 32-mm pellets of a European Commission Institute for Reference Materials and Measurements Natural Moroccan phosphate standard (BCR-032 #919), was analyzed frequently during analysis routines to ensure calibration accuracy. Samples were measured directly without any preparation. Mineral compositions were determined using a Panalytical X’Pert Pro θ/θ diffractometer equipped with Cu Kα source (1.5406 Å) operating at 45 kV and 40 mA. A PixCel detector was used, and the data were collected in transmission mode with a 2D detector. An incident beam PreFIX module with an X-ray mirror for Cu radiation was used to allow non-destructive analysis.
Data source location	The data used in this set come from the three main Iberian variscite mining areas and from 15 sites in the Iberian Peninsula and France. The geoarchaeological samples have been obtained in different research, excavation and survey campaigns by different members of the research team, as well as from visits to different academic centres and museums over more than a decade.The spatial coverage of the whole set is delimited betweenNorthern boundary: 47,6100,000 −1290,556Southern boundary: 36,8167 −5636,684Eastern boundary: 41,7850,979 2,9025Western boundary: 42,25,575.62 67,771,41The data from French archaeological sites have been published by the authors in [1]. We present the secondary data processed in the repositoryThe data from the north-east of the Peninsula come from specimens from the excavations and investigations carried out by (MEiB and FB) as well as from the following centers and museums: • Museu Nacional de Arqueología de Catalunya,Barcelona, Spain • Seminari d’Estudis I Recerques Prehistòriques,University of Barcelona, Spain The data from the south of the Iberian Peninsula come from the research of (CO and JAGC) as well as from specimens found in museums:
	• Museo de Huelva, Huelva, Spain • Departamento de Prehistoria y Arqueología Universidad de Sevilla, Spain The data from the Spanish north and central plateau come from the research of (CO, RVG, JAG, RBB, PB) as well as from specimens in the following museums:Museo de Ávila, Ávila, SpainMuseo de Salamanca, Salamanca, SpainMuseo de Segovia, SpainMuseo de Zamora,Museo arqueológico provincial de Palencia, SpainMuseo de Santa cruz, Toledo, Spain
Data accessibility	Repository name: Zenodo Data identification number: 10.5281/zenodo.15130535. Direct URL to data: https://zenodo.org/records/15131338
Related research article	Sanchez-Gomez D, Garrido-Cordero JÁ, Martínez-Blanes JM, García RV, Sousa AC, Vega MDZ, et al. A Forest of Green Beads: A Machine-Learning Based Framework to Determine the Geological Provenance of Prehistoric Variscite Artifacts. Rochester, NY: Social Science Research Network; 2025. doi: 10.2139/ssrn.5214878

## 1 Value of the Data

• This dataset compiles the largest geoarchaeological sample to date (*n* = 1778) from three major Iberian variscite deposits: Aliste (Zamora), Encinasola (Huelva), and Gavà Mines (Catalonia)—significantly expanding previous datasets
• This resource aims to fill critical gaps in the compositional uncertainty and geological provenance of prehistoric green phosphate beads.
• As a supervised ensemble, it is of great value as it can be used to train different computational models; be used as validation for existing models, to perform different types of statistical and social networks analysis, reference, teaching or collaboration in the study of European prehistoric mineral personal adornments.
• The data and code are freely accessible through public repositories, ensuring compliance with FAIR and Open Science practices.

## 2 Background

This dataset is part of a larger study [2] that addresses critical gaps in the provenance analysis of variscite and related green phosphate minerals, which serve as key tracers of prehistoric socio-economic networks in Late Prehistoric Europe (c. 6000–1200 BCE).

Despite decades of research, provenance studies still rely on a remarkably small dataset—approximately 192 samples from four deposits in Spain and France (Supplementary). This limited sample forms the basis for all archaeological models and assumptions regarding the distribution of this proxy, yet it fails to reflect the true complexity of the deposits. As a result, key geological variations remain underrepresented, raising concerns about the reliability of provenance models and the accuracy of archaeological narratives built upon them.

## 3 Data Description

**Fig. 1:**
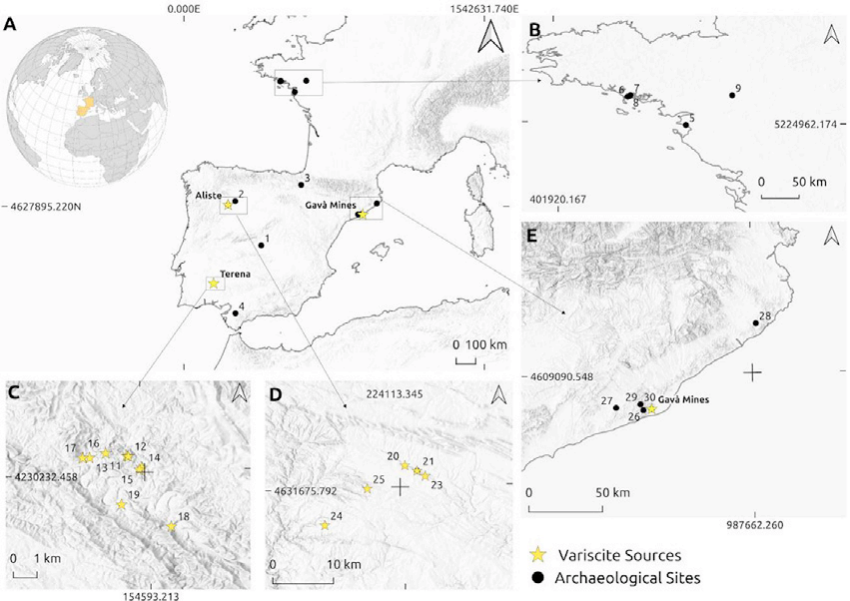
Location maps of sites, green phosphate sources and sub-sources in the dataset: **A:** Main Iberian phosphate sources; Aliste, the Gavà Mines, Encinasola; 1) Valle de las Higueras, 2) Las Peñas de Quiruelas, 3) Paternanbidea, 4) Alberite Dolmen. **B.** 5) La Josseliére, 6)Tumulus Mont-Saint-Michel, 7) Luffang, 8) Kervilor, 9) Auvernè, **C.** 10) Pico Centeno mine 1, 11) Pico Centeno mine 2, 12) Pico Centeno mine 3, 13) Los Barreros, 14) Sierra Concha 1, 15) Sierra Concha 2, 16) El tejar, 17) Los Barrancos, 18) La Lapa, 19) La Carvajera. **D.** 20) Peñas Mayas, 21) Las Cercas, 22) Peña el Sierro, 23) La Cogolla SE, 24) El Bostal, 25) Altos de la Vaca. **E**. 26) Cova Cassimanya, 27) La Serreta, 28) Cova Can Sadurní, 29) Roca de L'ivet, 30) Can Gambús *I*>.

The data presented here are the result of more than a decade of research by various members of the team (Table 1 and Fig. 1).

The dataset consists of five files in .xlsx and .odt format which are described below:

File name: VORTEX_dataset_references.odt
Description: Relevant literature for the data presented
File name: VORTEX_training_data.xlsx
Description: Geochemical data used for training a Random Forest supervised model
File name: VORTEX_real_world_data.xlsx
Description: Out-of-sample archaeological data used to test the model
File name: VORTEX_GeoSpatial_data.xlsx
Description: Geographical co-ordinates of archaeological sites and sample collection locations
File name: VORTEX_SPD(C14).xlsx
Description: Radiocarbon data available for the archaeological sites in the dataset
——————
File Description
——————

**VORTEX_training_data.xlsx:**

The file is presented in excel spreadsheet format with the following tabs: Raw, Processed, TrainingData(Resampled),pXRF_LoD, (BCR-032#919)ReferenceMaterial and Ref_Material_Stats:

**VORTEX_training_data.xlsx[raw]:**

**Table:** 

Feature	Data_type	Feature_description
ID	String	Primary Key
Site	String	Response variable with three classes
Context(0–2)	String	Subsource sample location
Type	String	type of sample: G: Geological A: Archaeological
XRD1	String	Main mineral phase label obtained by means of XRD
XRD2-XRD4	String	Secondary mineral phase label obtained by means of XRD (optional)
Method	String	XRF quantification method
DateXRF	Date	Date of XRF analysis
Duration	Float	p-XRF sample analysis time
Mg to Th	Float	Predictor features: at %. per chemical element obtained by means of p-XRF
±	Float	Precision

**VORTEX_training_data.xlsx[Processed(100** **%)]:**

**Table:** 

Feature	Data_type	Feature_description
**ID**	String	Primary Key
Site	String	Response variable with three classes
Al to Th	Float	Predictor features: at %. per chemical element obtained by means of p-XRF
Σ	Float	Closure of compositional quantities (100 %)

**VORTEX_training_data.xlsx[TrainingData(Resampled)]:**

**Table:** 

Feature	Data_type	Feature_description
Al to Th	Float	Predictor features: at %. per chemical element obtained by means of p-XRF
Site	String	Response variable with three classes

**VORTEX_training_data.xlsx[pXRF_LoD]:** This tab reports the Limits of Detection (LoD’s) of the instrument, and the values used to impute below detection limit values.

**Table:** 

Feature	Data_type	Feature_description
Element	String	Symbol of chemical features from Mg to Th
Measure time	String	Measurement time for which the Limit of Detection is present (LoD). LOD's improve as a function of the square root of the testing time
LoD ppm	Integer	LoD is specified for each matrix in three sigma 99.7 % confidence level
LoD at %	Float	Lod as at %.
LoD/√2	Float	Imputation values for BDL

**VORTEX_training_data.xlsx[Reference Material]:** This tab contains the raw XRF data of the BCR032 Moroccan Phosphate Reference material. Values are presented in wt %.

**Table:** 

Feature	Data_type	Feature_description
Name	String	Key field: Unique identifier
Duration	Float	p-XRF sample analysis time
Mg to Th	Float	Elemental concentration in wt % per chemical element obtained by means of p-XRF.
±	Float	Standard deviation (precision) reported per each value in the previous column

**VORTEX_training_data.xlsx[Ref_Material_Stats]:**

This tab contains the descriptive statistics of the Reference Material Morocco Phosphate BCR032, comparative information between the reference values and the measurements obtained as well as correction factors for transforming Oxides into elements as the reference values are reported as oxides. The reference values are obtained from https://crm.jrc.ec.europa.eu/p/40455/40468/By-material-matrix/Other-manufactured-material s/BCR-032-MOROCCAN-PHOSPHATE-ROCK-trace-elements/BCR-032

**VORTEX_real_world_data.xlsx:**

This file presents the compositional data of 571 archaeological variscite artefacts used to test the model. The file has two types of tabs:

**VORTEX_real_world_data.xlsx[Site_raw]:**

**Table:** 

Feature	Data_type	Feature_description
ID	String	Primary Key
Site	String	Response variable with three classes
Mg to Th	Float	Predictor features: at %. per chemical element obtained by means of p-XRF
±	Float	Precision

**VORTEX_real_world_data.xlsx[Site]**

**Table:** 

Feature	Data_type	Feature_description
ID	String	Primary Key
Mg to Th	Float	Chemical characterisation obtained by p-XRF expressed in at %. 100 % closed

The data for the French sites have been published by [1] and only their conversion to At % is presented.

**VORTEX_GeoSpatial_data.xlsx:**

**Table:** 

Feature	Data_type	Feature_description
Site	String	Name of Archaeological Site
x_coord	Float	Geographical coordinate (Longitude)
y_coord	Float	Geographical coordinate (Latitude)

**VORTEX_GeoSpatial_data.xlsx[AlisteSubSources]:**

**Table:** 

Feature	Data_type	Feature_description
ID	String	Primary key of each of Aliste samples
Site	String	Name of the specific location of sample collection
X	Float	Geographical coordinate (Longitude)
Y	Float	Geographical coordinate (Latitude)

**VORTEX_GeoSpatial_data.xlsx[EncinasolaSubsources]:**

**Table:** 

Feature	Data_type	Feature_description
Site	String	Name of the specific location of sample collection
x_coord	Float	Geographical coordinate (Longitude)
y_coord	Float	Geographical coordinate (Longitude)

**VORTEX_SPD(C14).xlsx:**

**Table:** 

Feature	Data_type	Feature_description
Lab_id	String	Laboratory id (Primary Key)
Site	String	Archaeological site
C14-BP	Integer	Uncalibrated date
C14-SD	Integer	Precision
ref	String	Bibliographical reference
Associated	Bool	Whether directed associated with sample

## 4 Experimental Design, Materials and Methods

This study analyzes 1778 geoarchaeological samples from three major Iberian phosphate deposits with documented prehistoric exploitation. The three areas differ markedly in their archaeological records:

• **Gavà Mines (Can Tintorer)**: A large underground mining complex dated to the first half of the 4th millennium BCE, with over 120 mine shafts documented across 200 hectares. Samples from Gavà include geological material, manufacturing debris (e.g., bead preforms), finished beads, and workshop refuse recovered *in situ*.
• **Zamora (Aliste) and Huelva (Terena)**: These areas exhibit scattered surface workings. Samples consist exclusively of geological material, some of which may derive from ancient extraction activity directly associated with mining structures.

The various fieldworks and excavations that have recovered the materials whose data comprise the dataset, span more than a decade of research (2011–2022) and describe in detail the methods and protocols for excavation and surface collection of the materials. Table 1 lists these works, whose procedures are adapted to the particularities of the different outcrops. Samples were randomly collected during fieldwork, prioritizing fragments ≥ 5 mm thick to exclude weathered crusts or minor veinlets. The chemical composition of the samples was analyzed using an Oxford Instrument XMET-7500 handheld XRF equipped with a Rhodium (Rh) tube, a silicon drift detector (SDD), and an automatic 5-position filter changer. Quantification was performed using the SOILS-LE program, which employs the fundamental parameter (FP) method. This method is particularly effective for analyzing a large number of elements, making it well-suited for this study [3]. The mean analysis time per sample was 60 s and chemical elements from Mg to Th were measured and quantified. A 32-mm pellets of a European Commission Institute for Reference Materials and Measurements Natural Moroccan phosphate standard (BCR-032 #919) ,^1^ was analyzed frequently during analysis routines to ensure calibration accuracy. Samples were measured directly without any preparation.

**Table 1:** Dataset composition.

Deposit	Location	Samples (n)	References
**Aliste/Palazuelo de las cuevas**	Zamora province	511	[[4], [5], [6]]
**Terena/Encinasola**	Huelva province	439	[7]
**The Gavà Mines**	Garraf Massif, Catalonia	828	[[3], [8]]

Mineral compositions of a subsample (*N* = 249; n_Aliste = 122, n_Terena = 47, n_Gavà = 80) were determined using a Panalytical X’Pert Pro θ/θ diffractometer equipped with Cu Kα source (1.5406 Å) operating at 45 kV and 40 mA. A PixCel detector was used, and the data were collected in transmission mode with a 2D detector. An incident beam PreFIX module with an X-ray mirror for Cu radiation was used to allow non-destructive analysis. The result spectra were compared with the ICDD reference database [9]

### 4.1 Steps

The data was Processed in spreadsheets software and jupyter lab environment as follows:

1. All elements were converted into atomic percentages.
2. Below detection limit values (BDL) were replaced with an informed guess (LoD/√2) [10]. To calculate the imputation values, we used the device´s limits of detection (LoD)^2^ for a SiO2 matrix and estimated their conversion from parts per million (ppm) to atomic percent.
3. The composition of each sample was closed to 100 %. The final set of chemical elements present in the data are: Al, Si, P, S, Cl, K, Ca, *Sc*, Ti, V, Cr, Mn, Fe, Co, Ni, Cu, Zn, Ga, Ge, As, Se, Br, Rb, Sr, Y, Zr, Nb, Mo, Ru, Ba, Ta, W, Au, Hg, Tl, Pb, Th

The development notebooks with all the code are available at [2]

## Limitations

Mineralogical analysis was carried out directly on the unprocessed geoarchaeological samples due to their heritage nature. XRD analyses were therefore carried out by selecting the flattest possible surface of the samples. Occasionally, the X-ray beam could be larger than the surface of the sample and, therefore, some widening of the peaks may have occurred due to the roughness of the sample and a slight misalignment of the sample. In any case, these complementary analyses still allow obtaining reliable mineralogical information in a quick and non-destructive way, without the need to prepare the sample.

## Ethics Statement

The authors have read and follow the ethical requirements for publication in Data in Brief and confirm that this work does not involve human subjects, animal experiments, or any data collected from social media platforms.

## CRediT Author Statement

**Daniel Sanchez-Gomez**: Writing – review & editing, Writing – original draft, Visualization, Validation, Supervision, Software, Project administration, Methodology, Investigation, Formal analysis, Data curation, Conceptualization, Visualization. **José Ángel Garrido-Cordero**: Writing – review & editing, Writing – original draft, Validation, Investigation, Data curation, Conceptualization. **José María Martínez-Blanes**: Writing – review & editing, Writing – original draft, Supervision, Investigation, Conceptualization. **Rodrigo Villalobos García**: Writing – review & editing, Writing – original draft, Validation, Methodology, Investigation, Formal analysis. **Manuel Edo i Benaiges**: Investigation, Data curation, Validation. **Ana Catarina Sousa**: Writing – review & editing, Writing – original draft, Methodology, Formal analysis, Data curation, Funding aquisition. **María Dolores Zambrana Vega**: Investigation. **Ferran Borrell**: Writing – review & editing, Writing – original draft, Methodology, Investigation. **Rosa Barroso Bermejo**: Writing – review & editing, Writing – original draft, Investigation, Resources. **Primitiva Bueno Ramírez**: Writing – review & editing, Investigation, Resources. **Carlos P. Odriozola**: Writing – review & editing, Writing original draft, Investigation, Funding acquisition, Formal analysis, Validation, Supervision, Project administration.

## Data Availability

ZenodoVORTEX (Variscite Origin Recognition Technology X-ray based) Data. A european geoarchaeological green phosphate compositional dataset (Original data).

## Appendix Supplementary materials

Image, application 1

## Footnotes

2 ^Measurement time for which the Limit of Detection (LOD) is present. LODs improve as a function of the square root of the test time. LoD is specified for each matrix^^at three sigma 99.7% confidence level.^

## Footnotes

1 ^The measurements of the reference standard are presented in the dataset^
