# Supplementary material for: VORTEX (Variscite origin recognition technology X-ray based) Data. A European geoarchaeological green phosphate compositional dataset
Source: Data Brief. 2025 Aug 6;62:111961. doi: 10.1016/j.dib.2025.111961 (PMC12363471; doi:10.1016/j.dib.2025.111961)
Supplement: Supplementary file 1 [file mmc1.docx]

Supplementary Information for:

**VORTEX (Variscite Origin Recognition Technology X-ray based) Data. A european geoarchaeological green phosphate compositional dataset.**

Daniel Sánchez-Gomez, José Ángel Garrido-Cordero, José María Martínez-Blanes, Rodrigo Villalobos García, Manuel Edo i Benaiges, Ana Catarina Sousa, María Dolores Zambrana Vega, Ferran Borrell, Rosa Barroso Bermejo, Primitiva Bueno Ramírez, Carlos P. Odriozola

[**1. Data used to date for provenance analysis of prehistoric variscite in Europe to date: 1**](#_z5j3uhvfonoy)

[**2. Contexts of the sites studied and collected in the dataset: 2**](#_vqazqpzgkp1d)

[**References: 12**](#_cbvkulp2i1ye)

# **Data used to date for provenance analysis of prehistoric variscite in Europe to date:**

[Table 1](https://docs.google.com/document/d/1o6IqwN4zN1-bUibFg4CTbUd0Kc7nDMwOH_g39CqqykI/edit?pli=1#table_samples). Summary of data used to provenance prehistoric veriscites to date.

| **Type** | **Reference** | **Aliste** | **Terena** | **Gavà** | **Total** |
| --- | --- | --- | --- | --- | --- |
| A | This paper^1^ | 511 | 439 | 828 | 1778 |
| G | [[1]](https://www.zotero.org/google-docs/?rBJlLZ) | 6 | - | - | 6 |
| G | [[2]](https://www.zotero.org/google-docs/?VEfg08)^2^ | - | - | 83 | 83 |
| A | [[3]](https://www.zotero.org/google-docs/?n5ssPm)^3^ | * | - | 11 | 11 |
| A | [[4]](https://www.zotero.org/google-docs/?9PnJEn) | * | - | 4 | 4^+^ |
| A | [[5]](https://www.zotero.org/google-docs/?j2MFkg) | * | - | 10^+^ | 30^+^ |
| G | [[6]](https://www.zotero.org/google-docs/?O30T1j) | 6^+^ | - | - | 6^+^ |
| G | [[7]](https://www.zotero.org/google-docs/?iB1x8C) | - | 7 | - | 7 |
| G | [[8]](https://www.zotero.org/google-docs/?OnvPsG) | *^+◊^ | - | - | 54*^+◊^ |
| A | [[9]](https://www.zotero.org/google-docs/?bCVEkr)^4^ | *^+◊^ | - | 8^+^ | 45^+^ |
| A | [[9]](https://www.zotero.org/google-docs/?zKffD7)^5^ | *^+◊^ | - | 8^+^ | 30^+^ |
| A | [[10]](https://www.zotero.org/google-docs/?uBSiEU) | ^+◊^ | ^◊^ | ^+◊^ | ^+◊^ |
| A | [[11]](https://www.zotero.org/google-docs/?oVEeb0) | 6^+^* | - | ^+^* | ^+^* |
| A | [[12]](https://www.zotero.org/google-docs/?LZJUdv) | 6*^+◊^ | 2*^+◊^ | 6*^+◊^ | 14*^+◊^ |
| A | [[13]](https://www.zotero.org/google-docs/?O9d0kQ) | * | * | * | * |
| A | [[14]](https://www.zotero.org/google-docs/?8xlINe) | * | * | * | * |
| A | [[15]](https://www.zotero.org/google-docs/?OYl3Ua) | * | 37 | * | 37 |
| A | Odriozola (2014) | 4 | 6^+^ | 1 | 11 |
| A | [[16]](https://www.zotero.org/google-docs/?bVtkQq) | 61^◊+^ | 60^◊+^ | 17^◊+^ | 138^◊+^ |
| A | [[17]](https://www.zotero.org/google-docs/?1E2r7r) | 87^◊+^ | 78^◊+^ | 27^◊+^ | 192^◊+^ |
| A | [[18]](https://www.zotero.org/google-docs/?TixeHP) | 81^+^ | 77^+^ | 27^+^ | 185^+^ |
| *only mean values reported; ^+^reported fully or partially in earlier publications; ^◊^only plots reported. | | | | | |

1 All the data reported in this dataset has been measured by us and not published previously.

2 Of the 83 analysed samples 38 were phosphates and 6 a mixture of phosphates and sulphates. The data was not reported in any form.

3 They acknowledge to have analysed 10 geological samples, however the only give provenience details for 4 monocrystalline variscite samples.

4 They acknowledge to have analysed 45 geological samples, however the only give provenience details for 8 monocrystalline variscite samples.

5 They acknowledge to have analysed 30 geological samples, however the only give provenience details for 8 monocrystalline variscite samples (the same as in Edo i Benaiges et al. (1995a).

# **Contexts of the sites studied and collected in the dataset:**

**Terena (Huelva, Spain):**

The Pico Centeno mining complex is located in the Sierra de Huelva on the border with Portugal. There was geological evidence of variscite [[7,19]](https://www.zotero.org/google-docs/?dSnMED) and three prehistoric trench mines had been documented in 1999 [[20]](https://www.zotero.org/google-docs/?n2SMn5), but the mining complex had remained unpublished until the 2010 incursion of [[15]](https://www.zotero.org/google-docs/?LpV2IN)

Since then, a systemic characterisation of the sources and products was begun in order to establish the exchange mechanisms and spatial patterns of mineral distribution [[21–23]](https://www.zotero.org/google-docs/?Sl6UWX). At the same time, a survey was proposed that would allow a first approximation to the extent of prehistoric variscite working in the Terena Synform.

The green aluminophosphate deposits in Huelva are associated with levels of lidites and/or cherts and occasionally with those of ampelitic shales, siliceous sericitic cinerites and siliceous shales of Silurian age, intercalated with the former [[7]](https://www.zotero.org/google-docs/?uui2yo). They are usually located in areas close to dipping faults, filling anamastatic fractures of the siliceous bedding rocks, given their particular paragenesis by direct deposition of phosphate groundwater along fissures in the aluminium-rich bedrock [[24]](https://www.zotero.org/google-docs/?qrFeYQ). These mineralisations have a characteristic translucent green colour and present a massive, compact, brecciated structure with a fine grain size [[7,19]](https://www.zotero.org/google-docs/?6vvQoJ)

The geological samples analysed in this work correspond to the following mining sites: Sierra de la Lapa (Encinasola) (n=4), Sierra Concha (Encinasola) (n=35), El Tejar (Encinasola) (n=17), Barrancos (Barrancos, Portugal) (n=3), La Carvajera (Encinasola) (n=4), Castillos de Torres (Cumbres de San Bartolomé) (n=15) and Pico Centeno mines 1 (n=11), 2 (n=343) and 3 (n=13)

**Aliste Outcrops (Zamora, Spain):**

The district of Aliste is located on the western edge of the Spanish Meseta Norte in the province of Zamora, on a series of Palaeozoic formations which form an uneven landscape of peneplains furrowed by small but deep valleys. Since the late 1960s, when a team of geologists discovered a variscite outcrop within the hill of Las Cercas (Palazuelo de las Cuevas), it has been known that ancient mining took place here [[1]](https://www.zotero.org/google-docs/?WK2MG4).

The surveys and excavations carried out by [[25]](https://www.zotero.org/google-docs/?eQqpA0) allowed to characterise several outcrops as well as evidence of prehistoric exploitation at different points in the area. The largest veins of green ore and those that have been most intensively exploited are the veins found in the hills of Las Cercas (Palazuelo de las Cuevas) and La Cogolla (Palazuelo de las Cuevas/San Vicente de la Cabeza)

During the field campaign, the researchers recovered ninety-six mining tools as well as artefacts in the process of manufacture that were produced from local raw materials such as quartzite. All tools without exception show wear from use, although in most cases their working parts were not completely worn.

Therefore, it is considered that such artefacts do not reflect other activities and that they were used in ancient mining from the beginning, in particular, these tools have been ascribed to the earliest known period of use of the extracted mineral: the Neolithic and Copper Age. [[25]](https://www.zotero.org/google-docs/?tysAae)

In this work, the samples analysed come from:

Urrieta Cabada (n=4), Peña Mayas (n=36), Las Cercas (n=78), Peña el Sierro (n=170), La Cogolla (n=186) and Depósitos de Agua (n=6) pertaining to the Pobladura de Aliste-Bercianos de Aliste 8-km long outcrop; and from El Bostal (Trabazos) (n=1), Altos de la Vaca (El Poyo/San Cristóbal de Aliste)(n=22), La Mazada (Gallegos del Campo)(n=5) and Los Palombares (El Poyo)(n=3).

**The Gavà neolithic Mines (Catalonia, Spain):**

The Gavà Mines are located on the right bank of the mouth of the Llobregat River, in the municipality of Gavà, some 25 km SW of the city of Barcelona. The Palaeozoic formations composed of Silurian, Devonian and Carboniferous materials form the basis of the Garraf limestone massif and of the mine site itself. The extent of the mining work was carried out in an area of approximately 250 hectares comprising three main areas of exploitation in the urban fringe of Can Tintorer, and the forest areas of Can Ferreres, Can Badosa and Rocabruna. The mining complex was intensively used in multiple galleries and chambers between the 5th and 3rd millennium BC and was used as a burial context even before ( 4450-4275 cal BC) the mining boom [[26]](https://www.zotero.org/google-docs/?yTdAK0)

The samples analysed in this work belong to the two main sectors of the complex, Can Tintorer (n=592), Can Ferreres (n=238)

**La Serreta (Vilafranca del Penedès, Catalonia):**

Is an open-air site composed of 89 structures dating from the Early Cardial Neolithic to the Bronze Age, as well as a few more recent structures. It is part of the so-called “campos de silos”. The structures containing inhumations amount to 5, however, we must add one more, which was probably looted, and 3 more which have some characteristics that are not very similar to the ones found at the site. which we define, in a preliminary way, as symbolic structures. All these structures were not arranged in a concentrated manner and, therefore, do not seem to form a necropolis.

The materials analysed in this study correspond to necklace beads of the strcutures 5, 22 and 60. [[27]](https://www.zotero.org/google-docs/?8vYOo2)

E-5 was a large ellipsoidal negative structure (maximum diameter c. 240 cm) with a niche centred at the base. It had a looting pit that removed and fractured the niche's closing slab and displaced the burial from its original position. Despite this, it had a strict anatomical connection, so we deduce that the looting took place shortly after the sealing of the structure. The inhumed person, an adult male, had two flint pieces at the level of the lower back. In addition, five coral and three variscite beads were recovered from inside the burial niche [[28,29]](https://www.zotero.org/google-docs/?OPyZku)

In E-22 was documented a necklace in primary position made of a tubular bead and 5 variscite discoidal beads, a pair of perforated Glycymeris sp. and, next to it, a honeyed flint flake.

E-60 is a small ellipsoidal structure with a very low preserved height of no more than 30 cm. Inside it were the remains of an adult male individual in left lateral decubitus. It contained a wide range of materials that correspond to the richest grave goods documented in the Penedès to date. It contained two polished axes and a set of 45 variscite beads on the right side of the skull (like a small sack).

**Cova Cassimanya (Baix Llobregat, Catalonia):**

On the south-west side of Les Agulles, at the Romagosa pass. Mossén Font i Sagué excavated the cave for the first time in 1899, finding human remains and archaeological material. Josep Mitjans, Josep Vendrell and archaeologist Pere Giró excavated it completely in the 1940s. As they explained, they found human and animal remains, fragments of coarse pottery (whiskers, flat and striated), gravel and flint flakes at very shallow depths, flint flake points (some with fins and peduncle and others lance-shaped, finely worked), bone sherds with V-shaped perforations, steatite, green phosphate (n=12) and bone beads (Giró, 1947-48). The materials are found at the museum of Vilafranca del Penedès [[30]](https://www.zotero.org/google-docs/?0kcmgN).

**Cova Can Sadurní (Baix Llobregat, Catalonia):**

The cave consists of a central room approximately 18 m long and 10 m wide, which extends into three small galleries totalling around 400 m2.

No other cave with five different prehistoric burial episodes is known in the whole of the Iberian Peninsula. The burial episode (5400 BC) of the Early Neolithic Cardial is the only burial necropolis of this period found to date in the Levante peninsular. The DNA analysis carried out on the 10 individuals found corroborates the presence of Near Eastern haplotypes and confirms the diffusionist theories of the Neolithization of the Western Mediterranean [[30,31]](https://www.zotero.org/google-docs/?FpNnKl).

The burial episode (4450-4200 BC) of the early Middle Neolithic is the closest detection of the funerary model used in the caves of this period in the north-east of the Iberian Peninsula. The discovery in the cave of all the tools and elements of the chain of exploitation and manufacture of variscite and green minerals during the early Middle Neolithic (4500-4000 BC) confirms the beginning of the mining of green phosphates during the second quarter of the 5th millennium at the hands of the pioneers of the site. [[32]](https://www.zotero.org/google-docs/?L4BjYe)

**Roca de l'Ivet cist (Llagostera, Catalonia):**

The Roca de L'Ivet cist consists of an oval-shaped funerary chamber, made up of 8 lateral supports and 3 frontal supports, measuring 2.10 metres long by 1.10 m wide, and oriented NW/SE.

Around the chamber there are remains of the existence of a circular tumulus, basically made of earth and of small dimensions (5-6 metres in diameter), which in the past covered the entire monument. At the same time, several small granite stones protrude from the ground at various points along its northern and western limits, which seem to have formed part of the remains of a possible outer ring of containment. It has been suggested that the monument dates from the end of the 4th millennium BC, which would make it one of the earliest megalithic manifestations of the Gavarres-Ardenya massif. [[33,34]](https://www.zotero.org/google-docs/?GL69Lo). For this study, n=36 beads found during the excavation of the compacted soil level inside the chamber were analysed.

**Valle de las Higueras (Huecas, Toledo):**

Is a necropolis formed by artificial caves dug into the top of the limestone hill. It is located in a valley in the Tagus river basin, which connects the Iberian hinterland with the Atlantic.[[35]](https://www.zotero.org/google-docs/?f5uzwZ)

The site represents an exceptional case for studying the dynamics surrounding the networks of green phosphate ornaments mainly for two main reasons:

Firstly, its inland location, far from the three provenance areas, certifies a remote origin of the materials, secondly, some of the assemblages were found in situ, with a clear association to individuals of whom it has been possible to establish their chronology.

The materials used in this study belong to:

Cave 1 (n= 211): Located in the western area, it consists of a chamber with a small niche and ante-chamber connected to it. A radiocarbon date was obtained from the human bone (Beta-145275: 3890 ± 40 BP) [[36]](https://www.zotero.org/google-docs/?pxVM0g). Cave 3 (Ante-chamber (3b) n=26, annex chamber (3c) n=35 ): This is located in the central area and consists of an ante-chamber, chamber and three niches in its walls. It contained at least 30 individuals. The beads formed part of the grave goods in the chamber and ante-chamber, but were absent in the niches.

Ante-chamber (3b): held at least 12 burials, some of which were secondary. Plain pottery, pieces of copper, flint arrowheads, bone pins, an amber bead, over a hundred shell beads (Trivia arctica) and 31 stone beads formed the grave goods. The adornments were concentrated next to two adults and a child. One of the individuals was dated by radiocarbon (Beta157732: 3830 ± 40 BP) [[36]](https://www.zotero.org/google-docs/?s3pzKK).

The annex chamber (3c), contained 11 individuals grouped on two oval-shaped stone floors and covered by cinnabar. An adult female located at the base (M-9) had 43 green beads in the area of her neck and a shell (Margarita auricularia) next to her hand. From her was obtained a date (Beta-205141: 3860 ± 40 BP)

Cave 4 (n=2): It had been destroyed at the time of the excavation. A small copper dagger, plain pottery and 2 green beads were found out of their original position.

Cave 7 (n=2): It consisted of two chambers dug on different heights. The East chamber contained a plain Bell Beaker pot with a burial dated to 3970±40 BP (Beta216245) [[36]](https://www.zotero.org/google-docs/?s1EhSD). At the back of the chamber osseous remains from a previous burial were associated with 2 flint arrowheads, a quadrangular stone pendant, nearly twenty black and white beads, and a fragment of copper

**Las Peñas (Quiruelas de Vidriales, Zamora):**

Las Peñas is located on the edge of a terrace that dominates the valley of the river Tera in the west of the Meseta Norte in the province of Zamora. It occupies an area of 2 ha and has a high concentration of prehistoric material such as pottery sherds, lithic and copper tools, and various variscite fragments such as plaques, beads in the process of being produced, and finished beads. It corresponds to a pre-Chalcolithic habitat, dated to around 2,500 BC [[25,37,38]](https://www.zotero.org/google-docs/?cb6Pzf)**.** During the field campaign of the surveys of the phosphate outcrops in the Aliste region [[25]](https://www.zotero.org/google-docs/?cTLCAk) excavated two test pits (1 and 2) on the north-eastern and south-eastern edges of the site, which in both cases revealed an intact prehistoric layer between 20 and 40 cm thick under the ploughsoil with abundant Copper Age material along with some variscite, as well as various fragments of daub with imprints of poles and a web of foliage which are probably remains of huts as identified on other Copper Age sites in the Meseta Norte.

**Can Gambús (Barcelona, Catalonia):**

Bòbila Madurell-Can Gambús is a Neolithic necropolis located in the valley known as the Vallés-Penedés Tectonic Trench, which separates the coastal and pre-coastal mountain ranges. The site is located in an area formed geologically by Quaternary sediments overlying conglomerates and clayey facies from the Upper Miocene. Most of the site is occupied by detrital material from the pre-coastal mountain range, composed of Quaternary red clay [[39]](https://www.zotero.org/google-docs/?pPXIPO). Bòbila Madurell-Can Gambús is the most emblematic cemetery on the cultural horizon of pit burials, due to the uniqueness of its archaeological record. A total of 179 burials have been documented, although it is estimated that this number may be higher. There is no similar Neolithic site in western Mediterranean Europe with so many tombs [[39,40]](https://www.zotero.org/google-docs/?SVt7Ku).

Since a motorway crossed the site in the middle, two main sectors can be distinguished: Bòbila-Madurell (southern part) and Can Gambús (northern part). Bòbila Madurell differs from Can Gambús because there are not only funerary structures, but also domestic ones, such as rubbish pits and silos. On the other hand, Can Gambús is located at the top of a hillside where only adult individuals were buried, while at Bòbila Madurell, at a lower altitude, both adult and immature individuals were documented.

At Can Gambús two zones are differentiated (Can Gambús I and Can Gambús II). This distinction was used in the excavation reports to differentiate four burials (located in Can Gambús II) that are slightly distant from Can Gambús I. In this work, n=78 beads from Can Gambús I were analysed; n=26 belong to the individual CG1-175 and n=52 to the individual CG1-246.

**Paternanbidea (Ibero, Pamplona):**

Paternanbidea is an open-air Neolithic site located in the municipality of Ibero in the Pamplona basin with an absolute dating of 6,090 ± 40 years BP and 5,960 ± 40 years BP obtained from bone fragments from two of the four pits (pit 1 and pit 2 respectively) that make up the burial site. Of the pits, three contain double burials (2, 3 and 4) and one with a multiple burial (pit 1) with at least five individuals. [[41–45]](https://www.zotero.org/google-docs/?S39PKC)

The site is of great importance in this work for several reasons: firstly, because of its strategic location in the north-east of the peninsula; secondly, because of its Neolithic chronology and, above all, because of the existence of a closed context, in which variscite beads have been found which, moreover, have a reliable radiometric dating. In this study we have analysed three beads from pit 2 in direct association with the individual PAT-2E2 and one bead from pit 1 with no direct association [[43]](https://www.zotero.org/google-docs/?apAFkA).

**Data from French sites:**

The data for the French megalithic monuments of Kervilor, Mané Bras (La Trinité-sur-Mer, Morbihan) n=35, Le Mont Saint-Michel (Carnac, Morbihan) n=3, Luffang, Tal er Roch (Crac'h, Morbihan) n=9, La Joselière (Pornic, Loire-Atlantique) n=9, Monts (Plichancourt, Marne) n=2, and Roche Mort (Grand-Auverné, Loire-Atlantique) n=3, have not been analysed by us. They are published in the volume [[46]](https://www.zotero.org/google-docs/?qMyJfh) and we have just preprocessed according to section 4.1 in order to adjust their format and use them as inputs.

According to [[18]](https://www.zotero.org/google-docs/?83QrNM), the chronology of these monuments ranges from 5000 BC in the case of Les Monts (Plichancourt) to 3000 BC in the case of Luffang, placing Saint Michel in the range 4700- 4300 BC, La Joselière and Kervilor between 4300 and 4000 BC.

The monument of Mané Bras, in the village of Kervilor, consists of two corridor tombs, one to the north of the cairn, with a quadrangular chamber (tomb 3), the other with a circular chamber (tomb 2). The corridors were generally oriented to the southeast, but separated by an angular drop of 13° [[47]](https://www.zotero.org/google-docs/?X5BBPw). The materials come from the corridor of the circular tomb where eight beads were collected under a cobblestone and from the northern chamber. The latter have been associated with arrowheads with finials and peduncles of bell-beaker ascription [[47]](https://www.zotero.org/google-docs/?WVjl41).

The tomb of La Joselière was built on the slope that descends towards the coastal cliff. It consists of four cells or chambers, symmetrically arranged on both sides of an axial corridor with an entrance facing southeast (Cassen et al., 2019). The data used in this paper come from n=9 beads from the south side chamber, at the foot of the presbytery slab. Although the pottery sherds collected in this chamber point unequivocally to the Middle Neolithic of the region (Auzay-Sandun), other equally fragmentary vessels attest to a late Neolithic use, which precludes drawing firm conclusions about the chronology of this site and proposing an extensive biography.

Mont Saint-Michel, the most imposing of the burial mounds in the Carnac region, has a trapezoidal cist with a low ceiling and walls made up of an alternation of elongated rough slabs and well agglomerated rubble. The access is blocked by other slabs placed at an angle to form the enclosure; the roof is provided by a granite slab split in two by the weight of the accumulations.

In this article we have only used data from n=3 of the more than 100 beads recovered in different excavation campaigns dating from the beginning of the 20th century. We do not have information on the specific context in which they were found, but we know that they are part of the impressive group of beads and pendants (n=128) of different materials; chalcedony, rock crystal and ‘ivory’, which were found accompanying the grave goods of the individual lying in the cist, with his head to the east, buried only in the lower level [[47]](https://www.zotero.org/google-docs/?6Tovq9).

On the banks of the Saulx, 7 km from its confluence with the Marne, the site of Les Monts (Plinchacourt) includes one or two Neolithic buildings revealed by post-holes and lateral construction pits. A group of three pit burials was also excavated 50 m from the domestic occupations, containing a woman, a man and a child, in a contracted position, with the limbs bent on the left side. The data of n=2 beads used in this work belong to burial 12 of a young woman buried in an oval pit with a strongly ochre-coloured fill in the vicinity of the skeletal bones with a date of (GrA- 17129: 6130 ±- 50 BP; 5200-4945), which confirms its ascription to the Early Neolithic [[47]](https://www.zotero.org/google-docs/?P6Y9ia)

The data of n=3 beads catalogued as belonging to Auvergne present a poor description involving a possible confusion of terms, pointed out by the authors of the sources consulted [[47]](https://www.zotero.org/google-docs/?8twp7l). It is not clear where these artefacts come from, whether from the Couronne Blanche dolmen on Petit Auverné or a cist in Roche Mort (Grand-Auverné).

The Tal er Roch megalithic burial site in the village of Luffang was initially explored in 1876 by M. Cappé and J. Miln. The monument had been critically affected by quarrymen who had reused the stone blocks, resulting in limited context. The data used corresponds to n=9 beads discovered in 1937 during a restoration operation at the site [[47]](https://www.zotero.org/google-docs/?Fac5Ds).

**Dolmen de Alberite (Villamartín, Cadiz):**

The megalithic structure of Alberite is located 4 km south of the municipality of Villamartín, dominating a valley floor, between the foothills of the Cadiz mountains, western slope, and the Guadalete river basin, to the east, forming an endorheic plain, with emerging flood deposits, foothills and slope spillways, controlling in the lower areas black earth formations and levels of Holocene silt-clay terraces, associated with the drainage of the Alberite stream. The Alberite I burial can be defined as a gallery dolmen with an entrance on the east side, whose chamber and long corridor form a single longitudinal space with a calibrated age of 4333-3977 and 4245- 3641, 2 sigma Cal. BC [[48,49]](https://www.zotero.org/google-docs/?ZdYWlT)**.** In this work we have analysed n=10 beads from inside the dolmen in an excavation level called the ochre level (-2.00 to -2.10 m). Although the beads have no direct association with dated individuals, they are part of an artefact assemblage that includes four silex slabs, 1,073 bone, shell and stone necklace beads, a quartz prism and a betilus idol [[49]](https://www.zotero.org/google-docs/?ktSFjB)

# **References:**

[1. Salvador P, Nicolau Ibarra JM, Martín Pozas JM, Arribas Moreno A, Galán Huertos E. Estudio mineralógico de la variscita de Palazuelo de las Cuevas, Zamora (España). Studia geologica salmanticensia. 1971; 115–132.](https://www.zotero.org/google-docs/?zQlF8s)

[2. Mata i Perelló JM, Plana F, Travería-Cros A. Estudio mineralógico del yacimiento de fosfatos de Gava. Boletín de la Sociedad Española de Mineralogía. 1983;7: 257–258.](https://www.zotero.org/google-docs/?zQlF8s)

[3. Blasco A, Edo Benaiges M, Villalba MJ. Les perles en callaïs du sud de la France proviennent-elles des mines de Can Tintorer ? Archéologie en Languedoc Congrès Hommage au Dr Jean Arnal. Colloque Int (20/09/1990):279–289. 1990. pp. 279–289.](https://www.zotero.org/google-docs/?zQlF8s)

[4. Edo Benaiges M, Blasco A, Villalba M. approche de la carte de distribution de la variscite de can Tintorer, Gavà (Catalogne). Cah Quat. 1990;17: 287–298.](https://www.zotero.org/google-docs/?zQlF8s)

[5. Villalba MJ, Edo Benaiges M, Blasco A. Zone d’influence de la calläis de Can Tintorer. Actes du colloque international de Nemours 1989. Paris; 1991. pp. 281–287. Available: https://www.researchgate.net/profile/Manel-Edo/publication/292146381_Zone_d%27influence_de_la_callais_de_Can_Tintorer/links/56aa650a08ae8f38656633db/Zone-dinfluence-de-la-callais-de-Can-Tintorer.pdf?origin=scientificContributions](https://www.zotero.org/google-docs/?zQlF8s)

[6. Fernández Turiel JL, Gimeno D, López Soler A, Querol Carceller X. Las mineralizaciones fosfáticas de los materiales paleozoicos de la provincia de Zamora. Anuario del Instituto de Estudios Zamoranos Florián de Ocampo. 1992; 463–506.](https://www.zotero.org/google-docs/?zQlF8s)

[7. Moro Benito MC, Gil Agero M, Montero Gómez JM, Cembranos Pérez ML, Pérez del Villar Guillén L, Fernández Fernández A, et al. Características de las mineralizaciones de variscita asociadas a los materiales silúricos del Sinforme de Terena, Encinasola (Provincia de Huelva). Comparación con las de la Provincia de Zamora. Boletín de la Sociedad Española de Mineralogía. 1992;15: 79–89.](https://www.zotero.org/google-docs/?zQlF8s)

[8. Moro Benito MC, Cembranos Pérez ML, Fernández Fernández A. 1995. Estudio mineralógico de las variscitas y turquesas silúricas de Punta Corveiro (Pontevedra, Españ). Geogaceta. 1995: 176–179.](https://www.zotero.org/google-docs/?zQlF8s)

[9. Edo Benaiges M, Villalba MJ, Blasco A. La Calaíta en la Península Ibérica. 1^o^ Congresso de Arqueologia Peninsular Actas VI. Sociedade portuguesa de Antropologia e Etnologia, Porto; 1995. pp. 127–168.](https://www.zotero.org/google-docs/?zQlF8s)

[10. Dominguez-Bella S, Morata D, de la Rosa J, Ramos J. Neolithic trade routes in SW Iberian Peninsula? Variscite green beads from some Neolithic sites in the Cadiz province (SW Spain): Raw materials and provenance areas. Proceedings of 32 International Symposium on Archaeometry 2000. Mexico, D.F. Instituto Investigaciones Antropológicas. Universidad Nacional Autónoma de México.; 2002.](https://www.zotero.org/google-docs/?zQlF8s)

[11. Villalba MJ. Le gîte de variscite de Can Tintorer: production, transformation et circulation du minéral vert. In: Guilaine J, editor. Matériaux, Productions, Circulations Du Neolithique à l’Age Du Bronze. 2002. pp. 115–130.](https://www.zotero.org/google-docs/?zQlF8s)

[12. Herbaut F, Querré G. La parure néolithique en variscite dans le sud de l’Armorique. Bulletin de la Société préhistorique française. 2004;101: 497–520. doi:10.3406/bspf.2004.13029](https://www.zotero.org/google-docs/?zQlF8s)

[13. Dominguez-Bella S. Variscite, a prestige mineral in the Neolithic-Aeneolithic Europe. Raw material sources and possible distribution routes. Slovak Geological Magazine. 2004;10: 147–152.](https://www.zotero.org/google-docs/?zQlF8s)

[14. Querré G, Herbault F, Calligaro T. Transport of Neolithic variscites demonstrated by PIXE analysis. X-Ray Spectrometry. 2008;37: 116–120.](https://www.zotero.org/google-docs/?zQlF8s)

[15. Odriozola CP, Linares-Catela JA, Hurtado-Pérez V. Variscite source and source analysis: testing assumptions at Pico Centeno (Encinasola, Spain). Journal of Archaeological Science. 2010;37: 3146–3157. doi:10.1016/j.jas.2010.07.016](https://www.zotero.org/google-docs/?zQlF8s)

[16. Querré G, Calligaro T, Domínguez-Bella S, Cassen S. PIXE analyses over a long period: The case of Neolithic variscite jewels from Western Europe (5th–3th millennium BC). Nuclear Instruments and Methods in Physics Research Section B: Beam Interactions with Materials and Atoms. 2014;318: 149–156. doi:10.1016/j.nimb.2013.07.033](https://www.zotero.org/google-docs/?zQlF8s)

[17. Querré G, Cassen S, Calligaro T. Témoin d’échanges au Néolithique le long de la façade atlantique: la parure en variscite des tombes de l’ouest de la France. In: Naudinot N, Meignen L, Binder D, Querré G, editors. Les systèmes de mobilité de la Préhistoire au Moyen Âge, Antibes, APDCA (Actes des rencontres inter- nationales d’archéologie et d’histoire d’Antibes. 2015. pp. 403–418.](https://www.zotero.org/google-docs/?zQlF8s)

[18. Querré G, Calligaro T, Cassen S. Origine des bijoux néolithiques en callaïs de l’ouest de la France. In: Querré G, Cassen S, Vigier E, editors. La parure en callaïs du Néolithique européen. Carnac: Archaeopress Publishing; 2019. pp. 129–199.](https://www.zotero.org/google-docs/?zQlF8s)

[19. Moro Benito MC, Gil Agero M, Montero Gómez JM, Cembranos Pérez ML, Pérez del Villar Guillén L, Fernández Fernández A, et al. Las mineralizaciones de variscita asociadas a los materiales volcano-sedimentarios silúricos del Sinforme de Terena, Encinasola (prov. de Huelva). Boletín de la Sociedad Española de Mineralogía. 1991;14: 101–102.](https://www.zotero.org/google-docs/?zQlF8s)

[20. Nocete Calvo F, Linares Catela JA. Las primeras sociedades mineras en Huelva: Alosno. Historia de la Provincia de Huelva,  Vol 1, 1999, ISBN 84-7156-341-X, págs 49-64. 1999. pp. 49–64. Available: https://dialnet.unirioja.es/servlet/articulo?codigo=4700127](https://www.zotero.org/google-docs/?zQlF8s)

[21. Odriozola CP, Linares-Catela JA, Hurtado-Pérez VM. Provenancing variscite beads: Pico Centeno (Encinasola, Spain) outcrop case study. Open J Archaeometry. 2013;1: 17. doi:10.4081/arc.2013.e17](https://www.zotero.org/google-docs/?zQlF8s)

[22. Odriozola CP, Villalobos García RV, Burbidge CI, Boaventura R, Sousa AC, Rodriguez-Ariza O, et al. Distribution and chronological framework for Iberian variscite mining and consumption at Pico Centeno, Encinasola, Spain. Quaternary Research. 2016;85: 159–176. doi:10.1016/j.yqres.2015.11.010](https://www.zotero.org/google-docs/?zQlF8s)

[23. Odriozola CP, Villalobos-García R. La explotación de variscita en el Sinforme de Terena: el complejo minero de Pico Centeno (Encinasola, Huelva). Trabajos de Prehistoria. 2015;72: 342–352. doi:10.3989/tp.2015.12158](https://www.zotero.org/google-docs/?zQlF8s)

[24. Larsen ES 3d. The mineralogy and paragenesis of the variscite nodules from near Fairfield, Utah, Part 1. American Mineralogist. 1942;27: 281–300.](https://www.zotero.org/google-docs/?zQlF8s)

[25. Villalobos García R, Odriozola CP. Organizing the Production of Variscite Personal Ornaments in Later Prehistoric Iberia: The Mines of Aliste and the Production Sites of Quiruelas de Vidriales (Zamora, Spain). European Journal of Archaeology. 2016;19: 631–651. doi:10.1080/14619571.2016.1147316](https://www.zotero.org/google-docs/?zQlF8s)

[26. Morell-Rovira B, Villalba MJ, Edo Benaiges M, Oms XF, Subirà ME, Santos FJ, et al. Cronología de las Minas neolíticas de Can Tintorer (Gavà, Barcelona): explotación minera y uso funerario. Munibe, Antropol -Arkeol. 2023 [cited 2 Oct 2024]. doi:10.21630/maa.2023.74.02](https://www.zotero.org/google-docs/?zQlF8s)

[27. Oms FX, Esteve X, Mestres J, Martín P, Gibaja JF, Torre MS de la, et al. La Serreta (Vilafranca del Penedès, Barcelona), un campo de silos entre el Neolítico Antiguo y la Edad del Bronce. SPAL - Revista de Prehistoria y Arqueología. 2024; 9–32. doi:10.12795/spal.2024.i33.01](https://www.zotero.org/google-docs/?zQlF8s)

[28. Borrello M, Bosch J, De Grossi J, Estrada A, Esteve X, Gorgoglione MA, et al. Les parures néolithiques de corail (Corallium rubrum L.) en Europa occidentale, Rivista di Scienze Preistoriche. Rivista di Scienza Preistoriche. 2012;LXII: 67–82.](https://www.zotero.org/google-docs/?zQlF8s)

[29. Esteve X, Oms FX, Martín P. Los enterramientos neolíticos de La Serreta (Vilafranca del Penedès, Barcelona. In: Gibaja J, Mozota M, Subirà ME, Martín A, editors. Mirando a la muerte Las prácticas funerarias durante el Neolítico en el noreste peninsular. Castelló de la Plana: e-DitARX Publicaciones Digitales; 2019. pp. 43–56.](https://www.zotero.org/google-docs/?zQlF8s)

[30. Villalba MJ, Blasco A, Edo Benaiges M. La prehitória al Baix LLobregat. Estat de la Qûestió. Castelldefels; 1989.](https://www.zotero.org/google-docs/?zQlF8s)

[31. Blasco A, Edo Benaiges M, Villalba MJ, editors. La cova de Can Sadurní i la prehistòria de Garraf. Actes de les Jornades Internacionals de PrehistòriaEl Garraf, 30 anys d’investigació arqueològica5 al 7 de desembre de 2008. Begues: EDAR-Hugony editore; 2011. Available: https://www.academia.edu/10906172/BLASCO_A_EDO_M_VILLALBA_M_J_eds_2011_La_cova_de_Can_Sadurn%C3%AD_i_la_prehist%C3%B2ria_de_Garraf_Actes_de_les_Jornades_Internacionals_de_Prehist%C3%B2ria_El_Garraf_30_anys_d_investigaci%C3%B3_arqueol%C3%B2gica_Begues_5_al_7_de_desembre_de_2008_Col_lecci%C3%B3_Actes_EDAR_Hugony_editore_Milano_2011_526_pp](https://www.zotero.org/google-docs/?zQlF8s)

[32. Edo Benaiges M, Antolín F, Martinez P, Villalba MJ, Fullola JM, Bergadá MM, et al. La cueva de Can Sadurní (Begues, Barcelona) : El episodio funerario del neolítico antiguo cardial pleno. Estado actual de la cuestión. In: Gibaja Bao JF, Subirà M, Martín A, Mozota M, Roig J, editors. Mirando a la Muerte: Las prácticas funerarias durante el neolítico en el noreste peninsular. E-ditArx; 2019. pp. 207–377.](https://www.zotero.org/google-docs/?zQlF8s)

[33. Niell Ciurana X. Intervenció arqueològica a la cista de la Roca de l’Ivet (Llagostera, Gironès). Universitat de Girona; 2014. Available: https://dugi-doc.udg.edu/handle/10256/13540](https://www.zotero.org/google-docs/?zQlF8s)

[34. Tarrús i Galter J, Chinchilla J. Els monuments megalítics. 1992. Available: https://dialnet.unirioja.es/servlet/libro?codigo=223320](https://www.zotero.org/google-docs/?zQlF8s)

[35. Barroso RM, Odriozola CP, Ramírez PB, García RV, Behrmann RB, Blanes JMM. MINERAL ADORNMENTS AT CHALCOLITHIC SITES IN INLAND IBERIA: VARISCITE BEADS AT VALLE DE LAS HIGUERAS (HUECAS, TOLEDO) SPAIN. Mediterranean Archaeology and Archaeometry. 2021;21: 177–177.](https://www.zotero.org/google-docs/?zQlF8s)

[36. Bueno P, Barroso RM, Behrmann R de B. Ritual campaniforme, ritual colectivo: la Necrópolis de cuevas artificiales del Valle de las Higueras, Huecas, Toledo. Trabajos de Prehistoria. 2005;62: 67–90. doi:10.3989/tp.2005.v62.i2.69](https://www.zotero.org/google-docs/?zQlF8s)

[37. Blanco Majado J, Martín Benito JI. El yacimiento calcolítico de Las Peñas en Quiruelas de Vidriales (Zamora). Brigecio: revista de estudios de Benavente y sus tierras. 1996; 11–30.](https://www.zotero.org/google-docs/?zQlF8s)

[38. Majado JB, Alonso MAL, Edo Benaiges M, Fernandez-Turiel JL. Estudio analítico de determinación mineralógica y de composición química de las cuentas de collar de calaíta y otras materias del yacimiento de Las Peñas (Quiruelas de Vidriales, Zamora). 1995.](https://www.zotero.org/google-docs/?zQlF8s)

[39. Gibaja JF, Morell B, Álvarez JAB, Duboscq S, Masclans A, Remolins G, et al. The Chronology of the Neolithic Necropolis Bòbila Maduell-Can Gambús in the Northeast Iberian Peninsula: Dating the Pit Burials Cultural Horizon and Long-Range Raw Material Exchange Networks. Radiocarbon. 2017;59: 1713–1736. doi:10.1017/RDC.2017.131](https://www.zotero.org/google-docs/?zQlF8s)

[40. Allièse F. Les sépultures de la Bòbila Madurell-Can Gambús (Vallès occidental). Éclairages sur les pratiques funéraires du nord-est de la péninsule Ibérique à la fin du V^e^ et au début du IV^e^ millénaire Tome 1 : texte Soutenue. TDX (Tesis Doctorals en Xarxa). Ph.D. Thesis, Universitat Autònoma de Barcelona. 2016. Available: https://www.tdx.cat/handle/10803/401004](https://www.zotero.org/google-docs/?zQlF8s)

[41. García Gazólaz J. Los enterramientos neolíticos del yacimiento de Paternanbidea (Ibero). Gobierno de Navarra. La Tierra te sea leve. Gobierno de Navarra. Pamplona; 2007. pp. 59–65. Available: https://www.culturanavarra.es/uploads/files/La%20Tierra%20te%20sea%20leve_2023(1).pdf](https://www.zotero.org/google-docs/?zQlF8s)

[42. Gazólaz JG. Paternanbidea (Ibero, Navarra): un yacimiento al aire libre de la Prehistoria reciente de Navarra. Cuadernos de Arqueología de la Universidad de Navarra. 1998;6: 33–48. doi:10.15581/012.6.27792](https://www.zotero.org/google-docs/?zQlF8s)

[43. Hervella M, Izaguirre N, ALONSO S, Fregel R, De La Rúa C. Enterramientos en fosa en el Neolítico Antiguo en Navarra: eva- luación de las evidencias arqueológicas mediante el estudio antro- pológico y molecular. Revista Española de Antropología Física. 2009; 31–38.](https://www.zotero.org/google-docs/?zQlF8s)

[44. Rojo Guerra M, GarcÍa MartÍnez de Lagrán I, Garrido Pena R, Tejedor C, Subirà De Galdàcano E, García Gazólaz J, et al. Enterramientos del Neolítico antiguo en el interior peninsular: nuevos datos para una actualización de la evidencia empírica. Del neolític a l’edat del bronze en el Mediterrani occidental: estudis en homenatge a Bernat Martí Oliver. Valencia: Diputación de Valencia; 2016. pp. 181–210.](https://www.zotero.org/google-docs/?zQlF8s)

[45. Sesma Sesma J. Un tipo de estructuras de combustión en asentamientos al aire libre de Navarra: los casos de Epertegi y Paternanbidea. Actas del III Congreso del Neolítico en la Península Ibérica: Santander, 5 a 8 de octubre de 2003, 2005, ISBN 84-8102-975-0, págs 559-568. Instituto Internacional de Investigaciones Prehistóricas de Cantabria; 2005. pp. 559–568. Available: https://dialnet.unirioja.es/servlet/articulo?codigo=1960670](https://www.zotero.org/google-docs/?zQlF8s)

[46. Querré G, Cassen S, Vigier E, editors. La parure en callaïs du Néolithique européen. Oxford (GB): Archaeopress Publishing; 2019.](https://www.zotero.org/google-docs/?zQlF8s)

[47. Cassen S, Boujot C, Charvet, A, Grimaud V, Le Maux N, Le Pennec LP, et al. La parure en callaïs (variscite et turquoise) au Néolithique, dans la moitié nord de la France. Corpus et contextes. In: Querré G, Cassen S, Vigier E, editors. La parure en callaïs du Néolithique européen. Oxford (GB): Archaeopress Publishing; 2019. pp. 255–310.](https://www.zotero.org/google-docs/?zQlF8s)

[48. Dominguez-Bella S, Ramos-Muñoz J. Pico Centeno prehistoric variscite mines (Encinasola, Huelva, SW Spain). La parure en callaïs du Néolithique européen. Carnac: Archaeopress Publishing; 2019. pp. 71–84.](https://www.zotero.org/google-docs/?zQlF8s)

[49. Ramos Muñoz JF, Giles Pacheco F. El Dolmen de Alberite (Villamartín). Aportaciones a las formas económicas y sociales de las comunidades neolíticas en el noroeste de Cádiz. Servicio de Publicaciones de la Universidad de Cádiz; 1996. Available: https://rodin.uca.es/handle/10498/26693](https://www.zotero.org/google-docs/?zQlF8s)
